# 2-Amino­anilinium 6-carb­oxy­picolinate monohydrate

**DOI:** 10.1107/S1600536811025050

**Published:** 2011-07-02

**Authors:** Yu-Tang Wang, De-Jiang Gao

**Affiliations:** a College of Food Science and Engineering, Northwest A & F University, Yang Ling 712100, People’s Republic of China; b College of Chemistry, Jilin University, Changchun 130012, People’s Republic of China

## Abstract

In the title compound, C_6_H_9_N_2_^+^·C_7_H_4_NO_4_^−^·H_2_O, one amino group of diamino­benzene is protonated while one carb­oxy group of pyridine-2,6-dicarb­oxy­lic acid is deprotonated. In the anion, the CO_2_ and CO_2_H groups make dihedral angles of 4.0 (5) and 8.7 (4)° with the pyridine ring. In the crystal, extensive N—H⋯O, N—H⋯N and O—H⋯O hydrogen bonds occur between anions, cations and water mol­ecules.

## Related literature

For related compounds, see: Andre *et al.* (2011 ▶); Blagden *et al.* (2008 ▶); Smith *et al.* (2000 ▶); Kapildev *et al.* (2011 ▶).
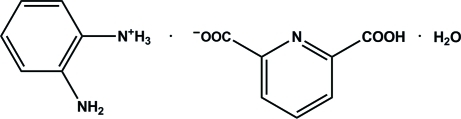


## Experimental

### 

#### Crystal data

C_6_H_9_N_2_^+^·C_7_H_4_NO_4_^−^·H_2_O
*M* *_r_* = 293.28
Monoclinic, 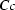

*a* = 12.408 (3) Å
*b* = 13.932 (3) Å
*c* = 8.0951 (16) Å
β = 102.41 (3)°
*V* = 1366.6 (5) Å^3^
*Z* = 4
Mo *K*α radiation
μ = 0.11 mm^−1^
*T* = 298 K
0.30 × 0.25 × 0.15 mm

#### Data collection

Rigaku Mercury2 diffractometer
Absorption correction: multi-scan (*CrystalClear*; Rigaku, 2005 ▶) *T*_min_ = 0.910, *T*_max_ = 1.000
7343 measured reflections
1555 independent reflections
1393 reflections with *I* > 2σ(*I*)
*R*_int_ = 0.041

#### Refinement

*R*[*F* ^2^ > 2σ(*F* ^2^)] = 0.040
*wR*(*F* ^2^) = 0.117
*S* = 1.12
1555 reflections
191 parameters
2 restraints
H-atom parameters constrained
Δρ_max_ = 0.19 e Å^−3^
Δρ_min_ = −0.22 e Å^−3^

### 

Data collection: *CrystalClear* (Rigaku, 2005 ▶); cell refinement: *CrystalClear* data reduction: *CrystalClear*; program(s) used to solve structure: *SHELXTL* (Sheldrick, 2008 ▶); program(s) used to refine structure: *SHELXTL*; molecular graphics: *SHELXTL*; software used to prepare material for publication: *SHELXTL*.

## Supplementary Material

Crystal structure: contains datablock(s) I, global. DOI: 10.1107/S1600536811025050/xu5250sup1.cif

Structure factors: contains datablock(s) I. DOI: 10.1107/S1600536811025050/xu5250Isup2.hkl

Supplementary material file. DOI: 10.1107/S1600536811025050/xu5250Isup3.cml

Additional supplementary materials:  crystallographic information; 3D view; checkCIF report

## Figures and Tables

**Table 1:** Hydrogen-bond geometry (Å, °)

*D*—H⋯*A*	*D*—H	H⋯*A*	*D*⋯*A*	*D*—H⋯*A*
N1—H1*A*⋯O1*W*^i^	0.89	2.13	3.004 (4)	166
N1—H1*B*⋯O3^ii^	0.89	1.98	2.862 (3)	170
N1—H1*C*⋯N3	0.89	2.12	3.003 (4)	171
N2—H2*A*⋯O1*W*^iii^	0.90	2.41	3.303 (4)	172
N2—H2*B*⋯O1	0.90	2.14	3.029 (4)	168
O1*W*—H1*WA*⋯O2^iv^	0.82	2.27	3.034 (3)	155
O1*W*—H1*WB*⋯O2	0.82	2.04	2.831 (3)	161
O4—H4⋯O1^i^	0.82	1.71	2.532 (3)	179

Symmetry codes: (i) 
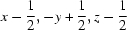
; (ii) 
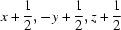
; (iii) 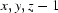; (iv) 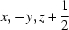.

**Fig. 1.**
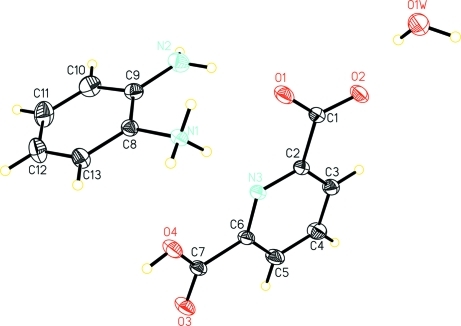
Molecular view of the title compound with the atomic numbering scheme. Displacement ellipsoids are drawn at the 30% probability level.

**Fig. 2.**
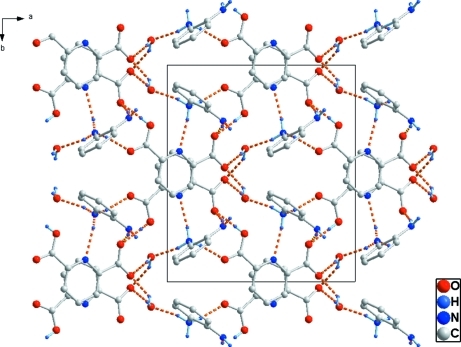
The crystal packing of the title compound viewed along the c axis showing the three-dimensionnal hydrogen bondings network (dashed line). Hydrogen atoms not involved in hydrogen bonding have been omitted for clarity.

## supplementary crystallographic information

### Comment

Cocrystals are most commonly thought of as structural homogeneous crystalline materials that contain two or more organic building blocks that are present in definite stoichiometric amounts. Within the development of pharmaceutical industry, molecular cocrystals are becoming increasingly important as a new drug with higher biomedical activity than the initial components. (Kapildev *et al.* 2011). Physicochemical properties such as the melting point, stability and solubility of an active pharmaceutical ingredient can be tuned through cocrystal formulation (Andre, *et al.* 2011; Blagden, *et al.* 2008; Smith, *et al.* 2000). These cocrystal forms often relie on the acid-amide H-bonds interactions. Herein, we report the crystal structure of the title compound, 2-aminoanilinium 6-carboxypicolinate monohydrate.

The asymmetric unit is composed of one 6-carboxypicolinate anion one 2-aminoanilinium cation and one water molecule (Fig.1). The amine N1 atom was protonated. And one of the carboxyl groups was deprotonated. The interplanar angle between the benzene and the pyridine rings equals to 88.89 (10)°. The geometric parameters of the title compound are in the normal range.

The molecular packing is stabilized by strong intermolecular N—H···O, N—H···N and O—H···O hydrogen bonds. The H-bonds link the molecules into a three-dimensional network (Fig. 2 and Tab. 1).

### Experimental

A mixture of pyridine-2,6-dicarboxylic acid (2.0 mmol), benzene-1,2-diamine (2.0 mmol) and 40 ml water were added into a 100 ml flask and refluxed for 5 h, then cooled and filtrated. The solution was evaporated slowly in the air. Colorless block crystals suitable for X-ray analysis were obtained after one week.

### Refinement

All H atoms attached to C atoms were fixed geometrically and treated as riding with C-H = 0.93 Å (aromatic) with *U*_iso_(H) = 1.2*U*_eq_(C). The H atoms bonded to N1, N2, O1W and O4 were located in a difference Fourier map, in the last stage of the refinement they were restrained with the H—N2 = 0.90, H—N1 = 0.89 and H—O = 0.82 Å. U_iso_(H)=1.5U_eq_(N1,O1W,O4) and U_iso_(H) = 1.2U_eq_(N2). As no significant anomalous scatterings, Friedel pairs were merged.

### Crystal data

**Table:**

C_6_H_9_N_2_^+^·C_7_H_4_NO_4_^−^·H_2_O	*F*(000) = 616
*M**_r_* = 293.28	*D*_x_ = 1.425 Mg m^−^^3^
Monoclinic, *C**c*	Mo *K*α radiation, λ = 0.71073 Å
Hall symbol: C -2yc	Cell parameters from 1555 reflections
*a* = 12.408 (3) Å	θ = 2.2–27.4°
*b* = 13.932 (3) Å	µ = 0.11 mm^−^^1^
*c* = 8.0951 (16) Å	*T* = 298 K
β = 102.41 (3)°	Block, colorless
*V* = 1366.6 (5) Å^3^	0.30 × 0.25 × 0.15 mm
*Z* = 4	

### Data collection

**Table:**

Rigaku Mercury2 diffractometer	1555 independent reflections
Radiation source: fine-focus sealed tube	1393 reflections with *I* > 2σ(*I*)
graphite	*R*_int_ = 0.041
Detector resolution: 13.6612 pixels mm^-1^	θ_max_ = 27.4°, θ_min_ = 2.2°
CCD profile fitting scans	*h* = −16→15
Absorption correction: multi-scan (*CrystalClear*; Rigaku, 2005)	*k* = −18→18
*T*_min_ = 0.910, *T*_max_ = 1.000	*l* = −10→10
7343 measured reflections	

### Refinement

**Table:**

Refinement on *F*^2^	Primary atom site location: structure-invariant direct methods
Least-squares matrix: full	Secondary atom site location: difference Fourier map
*R*[*F*^2^ > 2σ(*F*^2^)] = 0.040	Hydrogen site location: inferred from neighbouring sites
*wR*(*F*^2^) = 0.117	H-atom parameters constrained
*S* = 1.12	*w* = 1/[σ^2^(*F*_o_^2^) + (0.0734*P*)^2^] where *P* = (*F*_o_^2^ + 2*F*_c_^2^)/3
1555 reflections	(Δ/σ)_max_ < 0.001
191 parameters	Δρ_max_ = 0.19 e Å^−^^3^
2 restraints	Δρ_min_ = −0.22 e Å^−^^3^

### Special details

**Table:**

Geometry. All esds (except the esd in the dihedral angle between two l.s. planes) are estimated using the full covariance matrix. The cell esds are taken into account individually in the estimation of esds in distances, angles and torsion angles; correlations between esds in cell parameters are only used when they are defined by crystal symmetry. An approximate (isotropic) treatment of cell esds is used for estimating esds involving l.s. planes.
Refinement. Refinement of F^2^ against ALL reflections. The weighted R-factor wR and goodness of fit S are based on F^2^, conventional R-factors R are based on F, with F set to zero for negative F^2^. The threshold expression of F^2^ > 2sigma(F^2^) is used only for calculating R-factors(gt) etc. and is not relevant to the choice of reflections for refinement. R-factors based on F^2^ are statistically about twice as large as those based on F, and R- factors based on ALL data will be even larger.

### Fractional atomic coordinates and isotropic or equivalent isotropic displacement parameters (Å^2^)

**Table:**

	*x*	*y*	*z*	*U*_iso_*/*U*_eq_	
O1W	0.9144 (2)	0.11509 (17)	0.8457 (3)	0.0468 (6)	
H1WA	0.9052	0.0737	0.9133	0.070*	
H1WB	0.8972	0.0905	0.7516	0.070*	
O1	0.75215 (19)	0.17288 (15)	0.4341 (3)	0.0456 (6)	
O2	0.80493 (18)	0.02830 (16)	0.5392 (3)	0.0412 (5)	
N3	0.56474 (19)	0.10218 (18)	0.2422 (3)	0.0286 (5)	
O3	0.30560 (19)	0.10298 (17)	−0.0480 (3)	0.0436 (6)	
O4	0.41055 (17)	0.22264 (15)	0.0827 (3)	0.0397 (5)	
H4	0.3599	0.2569	0.0337	0.060*	
N1	0.61623 (19)	0.31257 (19)	0.2363 (3)	0.0296 (5)	
H1A	0.5582	0.3434	0.2587	0.044*	
H1B	0.6772	0.3318	0.3080	0.044*	
H1C	0.6076	0.2497	0.2476	0.044*	
C8	0.6256 (2)	0.33386 (19)	0.0621 (3)	0.0290 (6)	
N2	0.7964 (2)	0.2434 (2)	0.1010 (4)	0.0452 (7)	
H2A	0.8342	0.2069	0.0413	0.054*	
H2B	0.7933	0.2178	0.2020	0.054*	
C1	0.7403 (2)	0.0831 (2)	0.4445 (4)	0.0304 (6)	
C6	0.4728 (2)	0.0640 (2)	0.1448 (4)	0.0293 (6)	
C7	0.3880 (2)	0.1321 (2)	0.0494 (3)	0.0296 (6)	
C3	0.6225 (3)	−0.0586 (2)	0.3262 (4)	0.0371 (7)	
H3A	0.6748	−0.0990	0.3909	0.044*	
C9	0.7137 (2)	0.2968 (2)	0.0015 (4)	0.0336 (6)	
C5	0.4515 (3)	−0.0335 (2)	0.1314 (4)	0.0392 (7)	
H5A	0.3870	−0.0566	0.0623	0.047*	
C13	0.5445 (3)	0.3890 (2)	−0.0392 (4)	0.0378 (7)	
H13A	0.4864	0.4131	0.0041	0.045*	
C4	0.5287 (3)	−0.0961 (2)	0.2238 (5)	0.0427 (8)	
H4A	0.5173	−0.1621	0.2167	0.051*	
C10	0.7174 (3)	0.3179 (3)	−0.1655 (5)	0.0471 (8)	
H10A	0.7756	0.2946	−0.2097	0.057*	
C2	0.6380 (2)	0.0403 (2)	0.3314 (4)	0.0289 (6)	
C12	0.5500 (3)	0.4080 (3)	−0.2044 (5)	0.0503 (9)	
H12A	0.4953	0.4444	−0.2731	0.060*	
C11	0.6366 (4)	0.3728 (3)	−0.2669 (5)	0.0549 (10)	
H11A	0.6409	0.3859	−0.3779	0.066*	

### Atomic displacement parameters (Å^2^)

**Table:**

	*U*^11^	*U*^22^	*U*^33^	*U*^12^	*U*^13^	*U*^23^
O1W	0.0461 (14)	0.0470 (14)	0.0444 (13)	−0.0005 (11)	0.0035 (10)	0.0032 (10)
O1	0.0368 (12)	0.0319 (12)	0.0563 (14)	−0.0070 (10)	−0.0164 (10)	0.0073 (11)
O2	0.0311 (11)	0.0399 (12)	0.0452 (12)	0.0016 (9)	−0.0085 (9)	0.0071 (10)
N3	0.0222 (10)	0.0302 (13)	0.0312 (11)	−0.0011 (9)	0.0014 (9)	0.0013 (10)
O3	0.0307 (12)	0.0408 (13)	0.0504 (14)	0.0011 (9)	−0.0108 (10)	−0.0029 (10)
O4	0.0296 (11)	0.0318 (10)	0.0500 (13)	0.0023 (8)	−0.0086 (9)	0.0000 (10)
N1	0.0244 (11)	0.0326 (12)	0.0292 (12)	0.0008 (9)	−0.0003 (9)	−0.0013 (10)
C8	0.0278 (14)	0.0293 (13)	0.0286 (14)	−0.0010 (11)	0.0035 (10)	−0.0011 (11)
N2	0.0343 (14)	0.0463 (16)	0.0553 (18)	0.0115 (12)	0.0103 (13)	−0.0012 (14)
C1	0.0246 (13)	0.0325 (14)	0.0321 (14)	−0.0003 (11)	0.0015 (10)	0.0035 (12)
C6	0.0217 (12)	0.0314 (14)	0.0331 (14)	−0.0010 (11)	0.0020 (10)	−0.0005 (12)
C7	0.0228 (13)	0.0366 (14)	0.0272 (13)	−0.0010 (11)	0.0008 (10)	−0.0015 (12)
C3	0.0298 (16)	0.0312 (15)	0.0480 (18)	0.0007 (12)	0.0033 (13)	0.0080 (13)
C9	0.0298 (14)	0.0314 (15)	0.0395 (16)	−0.0014 (12)	0.0072 (12)	−0.0035 (12)
C5	0.0291 (16)	0.0372 (15)	0.0461 (18)	−0.0059 (13)	−0.0032 (13)	−0.0018 (14)
C13	0.0357 (15)	0.0397 (16)	0.0369 (17)	0.0079 (13)	0.0054 (12)	0.0033 (13)
C4	0.0363 (16)	0.0275 (15)	0.060 (2)	−0.0048 (13)	0.0016 (15)	0.0003 (14)
C10	0.049 (2)	0.053 (2)	0.045 (2)	0.0026 (16)	0.0206 (16)	−0.0055 (16)
C2	0.0228 (13)	0.0303 (14)	0.0320 (13)	−0.0012 (11)	0.0026 (11)	0.0021 (12)
C12	0.057 (2)	0.053 (2)	0.0387 (18)	0.0148 (18)	0.0064 (15)	0.0112 (16)
C11	0.072 (3)	0.061 (2)	0.0357 (19)	0.008 (2)	0.0202 (17)	0.0082 (17)

### Geometric parameters (Å, °)

**Table:**

O1W—H1WA	0.8201	C1—C2	1.518 (4)
O1W—H1WB	0.8201	C6—C5	1.384 (4)
O1—C1	1.264 (4)	C6—C7	1.502 (4)
O2—C1	1.245 (4)	C3—C4	1.378 (5)
N3—C2	1.345 (4)	C3—C2	1.391 (4)
N3—C6	1.349 (4)	C3—H3A	0.9300
O3—C7	1.219 (3)	C9—C10	1.394 (5)
O4—C7	1.307 (4)	C5—C4	1.390 (5)
O4—H4	0.8201	C5—H5A	0.9300
N1—C8	1.470 (4)	C13—C12	1.379 (5)
N1—H1A	0.8900	C13—H13A	0.9300
N1—H1B	0.8900	C4—H4A	0.9300
N1—H1C	0.8900	C10—C11	1.382 (6)
C8—C13	1.386 (4)	C10—H10A	0.9300
C8—C9	1.390 (4)	C12—C11	1.373 (6)
N2—C9	1.378 (4)	C12—H12A	0.9300
N2—H2A	0.9002	C11—H11A	0.9300
N2—H2B	0.8998		
			
H1WA—O1W—H1WB	106.3	C2—C3—H3A	120.4
C2—N3—C6	116.7 (2)	N2—C9—C8	122.5 (3)
C7—O4—H4	110.7	N2—C9—C10	120.3 (3)
C8—N1—H1A	109.5	C8—C9—C10	117.1 (3)
C8—N1—H1B	109.5	C6—C5—C4	118.4 (3)
H1A—N1—H1B	109.5	C6—C5—H5A	120.8
C8—N1—H1C	109.5	C4—C5—H5A	120.8
H1A—N1—H1C	109.5	C12—C13—C8	119.9 (3)
H1B—N1—H1C	109.5	C12—C13—H13A	120.0
C13—C8—C9	121.6 (3)	C8—C13—H13A	120.0
C13—C8—N1	118.8 (3)	C3—C4—C5	118.8 (3)
C9—C8—N1	119.6 (2)	C3—C4—H4A	120.6
C9—N2—H2A	113.6	C5—C4—H4A	120.6
C9—N2—H2B	125.0	C11—C10—C9	121.4 (3)
H2A—N2—H2B	113.1	C11—C10—H10A	119.3
O2—C1—O1	125.4 (3)	C9—C10—H10A	119.3
O2—C1—C2	118.4 (3)	N3—C2—C3	123.1 (3)
O1—C1—C2	116.3 (2)	N3—C2—C1	116.9 (2)
N3—C6—C5	123.8 (3)	C3—C2—C1	120.0 (3)
N3—C6—C7	117.6 (2)	C11—C12—C13	119.6 (3)
C5—C6—C7	118.6 (3)	C11—C12—H12A	120.2
O3—C7—O4	124.6 (3)	C13—C12—H12A	120.2
O3—C7—C6	121.3 (3)	C12—C11—C10	120.3 (3)
O4—C7—C6	114.1 (2)	C12—C11—H11A	119.8
C4—C3—C2	119.1 (3)	C10—C11—H11A	119.8
C4—C3—H3A	120.4		
			
C2—N3—C6—C5	−0.6 (4)	C6—C5—C4—C3	0.7 (5)
C2—N3—C6—C7	177.7 (3)	N2—C9—C10—C11	−178.8 (4)
N3—C6—C7—O3	176.1 (3)	C8—C9—C10—C11	−0.5 (5)
C5—C6—C7—O3	−5.4 (4)	C6—N3—C2—C3	0.1 (4)
N3—C6—C7—O4	−4.8 (4)	C6—N3—C2—C1	−178.6 (3)
C5—C6—C7—O4	173.7 (3)	C4—C3—C2—N3	0.8 (5)
C13—C8—C9—N2	178.7 (3)	C4—C3—C2—C1	179.4 (3)
N1—C8—C9—N2	−2.8 (4)	O2—C1—C2—N3	174.0 (3)
C13—C8—C9—C10	0.4 (4)	O1—C1—C2—N3	−5.8 (4)
N1—C8—C9—C10	178.9 (3)	O2—C1—C2—C3	−4.7 (4)
N3—C6—C5—C4	0.2 (5)	O1—C1—C2—C3	175.5 (3)
C7—C6—C5—C4	−178.1 (3)	C8—C13—C12—C11	−0.6 (6)
C9—C8—C13—C12	0.2 (5)	C13—C12—C11—C10	0.6 (6)
N1—C8—C13—C12	−178.4 (3)	C9—C10—C11—C12	0.0 (7)
C2—C3—C4—C5	−1.2 (5)		

### Hydrogen-bond geometry (Å, °)

**Table:**

*D*—H···*A*	*D*—H	H···*A*	*D*···*A*	*D*—H···*A*
N1—H1A···O1W^i^	0.89	2.13	3.004 (4)	166
N1—H1B···O3^ii^	0.89	1.98	2.862 (3)	170
N1—H1C···N3	0.89	2.12	3.003 (4)	171
N2—H2A···O1W^iii^	0.90	2.41	3.303 (4)	172
N2—H2B···O1	0.90	2.14	3.029 (4)	168
O1W—H1WA···O2^iv^	0.82	2.27	3.034 (3)	155
O1W—H1WB···O2	0.82	2.04	2.831 (3)	161
O4—H4···O1^i^	0.82	1.71	2.532 (3)	179

Symmetry codes: (i) *x*−1/2, −*y*+1/2, *z*−1/2; (ii) *x*+1/2, −*y*+1/2, *z*+1/2; (iii) *x*, *y*, *z*−1; (iv) *x*, −*y*, *z*+1/2.
